# 481. Trends in SARS-CoV-2-related Pediatric Hospitalizations in the Canadian Nosocomial Infection Surveillance Program, March 2020 to December 2022

**DOI:** 10.1093/ofid/ofad500.551

**Published:** 2023-11-27

**Authors:** Diane Lee, Robyn Mitchell, Linda Pelude, Charles Frenette, Bonita Lee, Marie-Astrid Lefebvre, Jeannette L Comeau, Jocelyn Srigley, Nisha Thampi

**Affiliations:** Public Health Agency of Canada, Ottawa, Ontario, Canada; PHAC, Ottawa, Ontario, Canada; PHAC, Ottawa, Ontario, Canada; McGill University Health Centre, Montreal, Quebec, Canada; University of Alberta, Edmonton, Alberta, Canada; Montreal Children’s Hospital, McGill University Health Centre, Montreal, Quebec, Canada, Montreal, QC, Canada; IWK Health Centre, Halifax, Nova Scotia, Canada, Halifax, NS, Canada; BC Women’s and BC Children’s Hospital, Vancouver, British Columbia, Canada; CHEO, Ottawa, Ontario, Canada

## Abstract

**Background:**

National surveillance can provide insights into trends in pediatric SARS-CoV-2-related hospitalizations during the pandemic and healthcare-associated infections.

**Figure:**
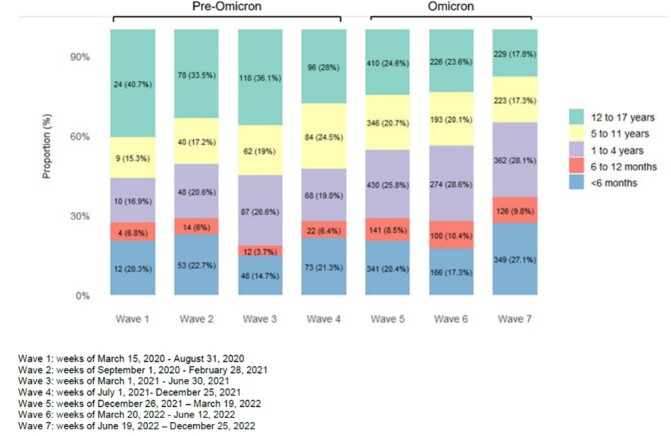
Proportion of all pediatric hospitalizations by age group and wave

**Methods:**

From March 1, 2020, to December 31, 2022, the Canadian Nosocomial Infection Surveillance Program collected patient-level data on pediatric patients (under age 18 years) hospitalized with laboratory-confirmed SARS-CoV-2 from 9 pediatric and 25 mixed adult-pediatric hospitals. Pediatric COVID-19 vaccines became available for 12-17 years in May 2021 and for 5-11 years in November 2021.

**Results:**

Of 4,878 pediatric patients, most (80.3%) were hospitalized with SARS-CoV-2 infection during the Omicron-dominant period (since Jan 2022); a higher proportion involved patients under five years of age (58.5% vs 46.9%, p < 0.001). Most hospitalizations pre-Omicron involved children not vaccinated against COVID-19 (92%) versus 70% during Omicron (p < 0.001). However, a lower proportion required intensive care during Omicron (15% vs 20%, p < 0.001). There was no difference in pre-existing comorbidities or mortality between periods.

Overall, there were 257 healthcare-associated COVID-19 infections (HA-COVID) reported (5.4%); 89% occurred during Omicron. While there was no difference in median ages of patients with HA-COVID and community-associated COVID-19 infections (CA-COVID), 62% of patients with HA-COVID had a pre-existing comorbidity compared to 44% with CA-COVID (p < 0.001), and 24% remained in hospital at 30 days after HA-COVID, compared to 2.2% with CA-COVID (p < 0.001). Nearly 50% of patients with HA-COVID had received at least one vaccine dose, compared to 25% with CA-COVID.

**Conclusion:**

During the Omicron-dominant period, a higher proportion of admitted patients with SARS-CoV-2 infection were under five years, but a lower proportion were unvaccinated and a lower proportion required intensive care and mortality was comparable between both periods. Only 5.4% of pediatric COVID-related hospitalizations were HAI, with most during Omicron. Patients with HAI were more likely to have a pre-existing comorbidity and increased hospital stay, potentially related to their underlying conditions.

**Disclosures:**

**All Authors**: No reported disclosures

